# Effects of Social Experience on the Habituation Rate of Zebrafish Startle Escape Response: Empirical and Computational Analyses

**DOI:** 10.3389/fncir.2018.00007

**Published:** 2018-02-05

**Authors:** Choongseok Park, Katie N. Clements, Fadi A. Issa, Sungwoo Ahn

**Affiliations:** ^1^Department of Mathematics, North Carolina A&T State University, Greensboro, NC, United States; ^2^Department of Biology, East Carolina University, Greenville, NC, United States; ^3^Department of Mathematics, East Carolina University, Greenville, NC, United States

**Keywords:** social status, habituation, Mauthner neuron, zebrafish, computational model, learning, aggression

## Abstract

While the effects of social experience on nervous system function have been extensively investigated in both vertebrate and invertebrate systems, our understanding of how social status differentially affects learning remains limited. In the context of habituation, a well-characterized form of non-associative learning, we investigated how the learning processes differ between socially dominant and subordinate in zebrafish (*Danio rerio*). We found that social status and frequency of stimulus inputs influence the habituation rate of short latency C-start escape response that is initiated by the Mauthner neuron (M-cell). Socially dominant animals exhibited higher habituation rates compared to socially subordinate animals at a moderate stimulus frequency, but low stimulus frequency eliminated this difference of habituation rates between the two social phenotypes. Moreover, habituation rates of both dominants and subordinates were higher at a moderate stimulus frequency compared to those at a low stimulus frequency. We investigated a potential mechanism underlying these status-dependent differences by constructing a simplified neurocomputational model of the M-cell escape circuit. The computational study showed that the change in total net excitability of the model M-cell was able to replicate the experimental results. At moderate stimulus frequency, the model M-cell with lower total net excitability, that mimicked a dominant-like phenotype, exhibited higher habituation rates. On the other hand, the model with higher total net excitability, that mimicked the subordinate-like phenotype, exhibited lower habituation rates. The relationship between habituation rates and characteristics (frequency and amplitude) of the repeated stimulus were also investigated. We found that habituation rates are decreasing functions of amplitude and increasing functions of frequency while these rates depend on social status (higher for dominants and lower for subordinates). Our results show that social status affects habituative learning in zebrafish, which could be mediated by a summative neuromodulatory input to the M-cell escape circuit, which enables animals to readily learn to adapt to changes in their social environment.

## Introduction

Most animals make context-dependent behavioral decisions as they navigate their environment (Calabrese, 2003; Kristan, 2008; Nienborg et al., 2012). For social animals these decisions are influenced in part by intraspecific social interactions that develop into long-term and stable dominance relationships (Issa et al., 2012; Miller et al., 2017). Different brain regions regulate different behaviors in social conflict (Chou et al., 2016). Although the effects of social experience on nervous system function has been investigated in both vertebrate and invertebrate systems, our understanding of how dominance relationships differentially affect learning remains limited (Yeh et al., 1996; Issa et al., 2012; Araki et al., 2013). One form of learning is habituative learning, a well-characterized form of non-associative learning during which an animal decreases its responsiveness to repeated stimuli (Thompson and Spencer, 1966; Rankin et al., 2009). While habituation has been described in many organisms including *Cnidarians* (Rushforth et al., 1964), aplysia (Kandel, 2009), crayfish (Krasne and Woodsmall, 1969; Araki et al., 2013), zebrafish (Eaton et al., 1977a; Marsden and Granato, 2015; Pantoja et al., 2016; Roberts et al., 2016) and humans (Davis, 1934), it remains unclear how social dominance leads to neural differences underlying habituation processes (Glanzman, 2009; Thompson, 2009).

Zebrafish has emerged as a good model system to investigate the neural mechanisms in behavioral neuroscience. When paired, zebrafish interact aggressively with conspecifics with aggressive displays that increase in intensity until a stable social hierarchy is established that persists for weeks (Larson et al., 2006; Oliveira et al., 2011; Pavlidis et al., 2011; Miller et al., 2017). In addition, zebrafish exhibit distinct simple behaviors that can be readily quantified behaviorally and whose neural correlates have been probed thoroughly (Eaton et al., 2001). These characteristics present an opportunity to study how short-term habituation is modulated by the long-term social experience on the activation dynamics of neural circuits.

**Figure 1:**
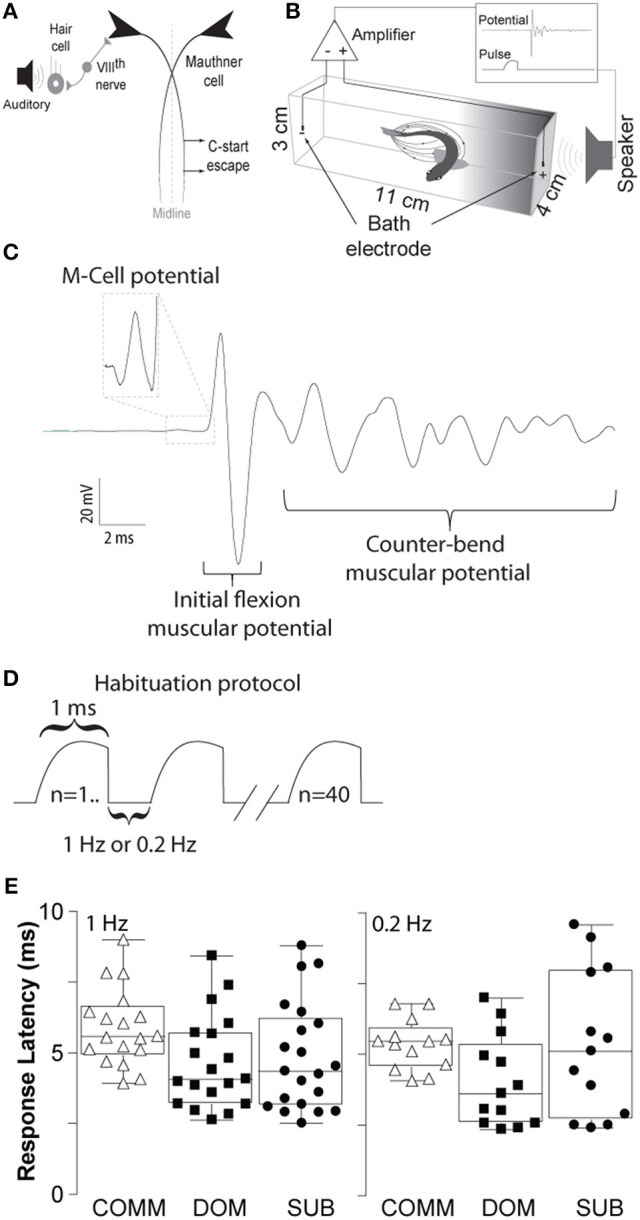
Schematic illustrations of the M-cell escape circuit, experimental setup and recording. **(A)** Schematic illustration of the M-cell escape circuit. Zebrafish startle response is activated by auditory stimuli. C-start behavior is mediated by the Mauthner neural circuit. M-cell innervates contralateral spinal cord motor neurons that activate the musculature. Activation of the M-cell is necessary for C-start escape. **(B)** A pair of bath electrodes is placed on each end of each testing chamber. Bath electrodes detect neuromuscular field potentials generated as the M-cell escape response is activated. M-cell escape is activated by an auditory pulse. Field potentials and stimuli are time-locked and digitally recorded. **(C)** An illustrative example of a phasic field potential recording during activation of the C-start escape response mediated by the M-cell followed by C-start bend and counter-bends. **(D)** Representation of the repeated auditory stimuli at 1 and 0.2 Hz. **(E)** Latency between the stimulus and the phasic potential for communals (COMM), dominants (DOM), and subordinates (SUB) at 1 and 0.2 Hz. Mean±SEM was plotted.

One of these neural circuits is the Mauthner neuron (M-cell) escape circuit that mediates the startle avoidance response (Eaton et al., 2001). An abrupt auditory pulse to the ear elicits a quick (<10 ms) and highly stereotypical startle escape response. The functional and anatomical organizations of the underlying neural circuit that mediates the startle escape response have been studied in larval and adult zebrafish, and goldfish (O'Malley et al., 1996; Eaton et al., 2001; Severi et al., 2014; Thiele et al., 2014; Wang and McLean, 2014). A distinct reticulospinal neural network centered around the M-cells is known to initiate and control the startle escape response (Figure 1A). The M-cells constitute a pair of neurons that receive ipsilateral synaptic sensory input (Sato et al., 2007; Kohashi and Oda, 2008; Mu et al., 2012) and project axons to innervate contralateral spinal cord motor neurons to elicit a rapid fast flexion (Eaton et al., 1977b; Zottoli et al., 1987; Canfield, 2003). Moreover, the startle escape response can be modulated by social status (Neumeister et al., 2010; Whitaker et al., 2011). The mechanism of modulation includes the excitability of the M-cell as well as excitatory and inhibitory drives to the M-cell escape circuit (reviewed in Medan and Preuss, 2014). In addition, Miller et al. (2017) have shown that the escape circuit is differentially activated in socially dominant and subordinate animals: dominant animals enhance activation of the swim circuit while subordinate animals swim less but their escape response is more sensitive.

Not only is the escape circuit prone to social plasticity, it is also known to be susceptible to habituation. Repeated auditory stimulation at a rate of 0.2 Hz rapidly decreases response probability (Marsden and Granato, 2015). Moreover, rate of habituation depends on the intensity as well as the frequency of stimulation (reviewed in Rankin et al., 2009). However, it is poorly understood how social status influences the habituation process of the M-cell startle escape response to repeated auditory stimulation. It is known though that the escape circuit is regulated by neuromodulatory inputs (Pereda et al., 1992; McLean and Fetcho, 2004). Neuromodulatory inputs that regulate the excitability of the escape circuit seem to influence the M-cell habituation rate to repeated stimuli. Marsden and Granato (2015) suggested a model for habituation as the combination of increased inhibitory input from feed-forward glycinergic inhibitory neurons and decreased excitatory input from auditory afferents, which together decrease the net excitability of the M-cell. Thus, the escape circuit may also be influenced by sustained social cues during social interactions that enable the animals to learn their social standing as they interact with conspecifics.

In the present study, we investigated a potential mechanism of how neuromodulatory inputs that are known to regulate the excitability of the M-cell escape circuit can be altered to mediate differences in habituation rates depending on the social status and characteristics of stimulation inputs. We constructed a simplified neurocomputational model of the M-cell escape circuit to test this idea and investigated how the interplay among social status, cellular excitability, and characteristics (frequency and amplitude) of repeated stimulations affects the habituation of the M-cell escape response.

## Materials and methods

### Animal maintenance

The experiments were approved by the East Carolina University Committee of Institutional Animal Care and Use. Wild type AB zebrafish strain (7–12 months old) (*Danio rerio*) were housed communally with mixed sex (~20 fish per 10 gallon tanks) at 28°C under a 14 h/10 h light/dark cycle and fed three times daily to satiation with a commercial food (Otohime B2, Reed Mariculture, CA, USA). Food was supplemented with newly hatched artemia (Brine Shrimp Direct, UT, USA).

### Social isolation, pairing and behavioral observations

Males were randomly selected from communal tanks and physically and visually isolated from conspecifics for 1 week in isolation tanks (23 × 13 × 6 cm). Following isolation, animals were randomly paired with a conspecific continuously for 2 weeks in a novel tank of equal dimension to the isolation tanks. To determine social status formation and stability of social relationships, we recorded the daily aggressive behavior between animals during 5 min of observations. Observations occurred in the morning between 10 a.m.−12 p.m. when animal activity was relatively elevated. We counted the total number of attacks/bites and retreats displayed by each animal. These behaviors are reliable measures of assessing social dominance as described previously (Oliveira et al., 2011). Social dominance was calculated by taking the ratio of the total number of aggressive to submissive behavior. The animal with a higher ratio is considered the dominant animal. In rare instances when the dominance relationship was unstable or reversed the pairs were excluded from the study (*n* = 2 out of 35). These paired animals quickly form stable dominance relationships by starting on the third day of interactions and remained stable for the remainder of the 2 weeks (Miller et al., 2017). As an experimental control we also tested male communal animals of similar age and size as the experimental animals. The communal fish were housed in mixed sex tanks of ~20 fish.

### Experimental setup

Animals were transferred to two separate but identical testing chambers (dimensions: 11 × 4 × 3 cm) and allowed to acclimate for a period of 30 min. With this experimental arrangement the animals were physically, visually and chemically separated. The chambers were equidistant from the speaker (4 cm) (Figure 1B). The testing chambers contained double distilled water with a resistance of ~15 MΩ-cm and temperature of 25°C. As previously described, high resistive water was found to improve the signal to noise ratio of the electrical field generated during escape behavior, and long-term exposure to water with low ionic concentration does not have obvious effects on the behavior or stress level of the animals (Issa et al., 2011; Monesson-Olson et al., 2014).

To record the electric field potentials of the escape response we used a pair of bipolar conductive electrodes (1 mm bare thickness, 3–5 mm metal exposure). Each pole was placed at the end of the testing chamber (Figure 1B). Electric signals were amplified 1,000-fold using an AC differential amplifier with a low cut-off at 300 Hz and high cut-off at 1 KH (AM-Systems model 1700, Carlsborg, WA, USA). Signals were digitized using a Digidata-1322A digitizer and stored using the Axoscope software (Molecular Devices, Inc., Sunnyvale, CA, USA). Identification of the M-cell mediated escape response can be reliably identified due to the short-latency and large amplitude of the field potential generated during escape. Onset of M-cell mediated escape occurs between 5 and 15 ms with a characteristically large and phasic electric field potential of the M-cell followed by the neuromuscular field potentials of motor neurons during escape (Figure 1C). Figure 1E shows the latencies for all three animal groups (dominants, subordinates, and communals) to consecutive 40 stimuli: communals had 5.73±0.25 ms (mean± SEM) at 1 Hz and 5.36±0.25 ms at 0.2 Hz; dominants had 4.65±0.37 ms at 1 Hz and 4.02±0.44 ms at 0.2 Hz; subordinates had 4.84±0.43 ms at 1 Hz and 5.36±0.72 ms at 0.2 Hz. Unlike the M-cell field potentials, the electric signal generated during swimming behavior is qualitatively and quantitatively distinct in that they are significantly smaller in amplitude and longer in duration compared to M-cell generated field potential (Prugh et al., 1982; Issa et al., 2011; Monesson-Olson et al., 2014). These features enable the unambiguous characterization of the field potentials generated by these two behaviors (Issa et al., 2011).

### Habituation trials

Auditory pulses were digitally generated via a computer using the Audacity open source audio editor and recorder software (www.audacityteam.org). The amplitude of auditory pulses was calibrated prior to the experiments using a decibel meter (Sinometer, MS6700). Supra-threshold auditory stimuli consisted of a phasic 1 ms at 95 dB re 20 μPa amplitude (sine wave). For the habituation experiments, we delivered 40 repeated stimuli at 1 or 0.2 Hz (Figure 1D).

### Data analysis

All statistical analyses were performed in MATLAB (Mathworks, Nautick, MA) and Prism (GraphPad software Inc., San Diego, USA). Unless specified otherwise, all comparisons were first subjected to analysis of variance testing (ANOVA) or mixed design ANOVA (a mixture of one between-group and repeated measures variables) followed by Tukey's HSD *post-hoc* test for all multiple comparisons.

### Neuronal model

In a previous study (Miller et al., 2017), a neurocomputational model of the escape and swim circuits in the zebrafish was constructed to study how social status may regulate the activation of the escape and swimming behaviors. In that model, M-cells were the main command neurons in the escape circuit and the stimulus was directly delivered unilaterally to one of the M-cells. In the present study, we used this neurocomputational model as a simplified M-cell escape circuit and tested one possible mechanism by which the rate of habituation of the escape circuit with respect to repeated external stimuli can be regulated by social status and characteristics of repeated stimulations.

The M-cell model used a conductance-based modified Morris-Lecar neuronal model (Morris and Lecar, 1981; Izhikevich, 2007; Ermentrout and Terman, 2010) with additional calcium-dependent potassium current. The membrane potential of each cell obeys the following current balance equation:

(1) $$\begin{array}{l} C\frac{dv}{dt}=\;-I_{Ca}-I_{K}-I_{L}-I_{KCa}-I_{syn}+I_{app}\left(t\right), \end{array}$$

where *I*_*K*_ = *g*_*K*_*n* (*v* − *v*_*K*_), *I*_*Ca*_ = *g*_*Ca*_ *m*_∞_(*v*)(*v* − *v*_*Ca*_), $I_{KCa}=g_{KCa}\;\left\{\frac{\left[Ca\right]}{\left[Ca\right]+k_{1}}\right\}\left(v-v_{K}\right),\;I_{L}=g_{L}\left(v-v_{L}\right)$ represent the potassium, calcium, calcium-dependent potassium, and leak currents, respectively. *m*_∞_ is an instantaneous voltage-dependent gating variable for the calcium current where:

(2) $$\begin{array}{l} m_{\infty }\left(v\right)=0.5\left(1+\tanh\left(\frac{v-v_{1}}{v_{2}}\right)\right). \end{array}$$

The concentration of intracellular *Ca*^2+^ is governed by the calcium balance equation:

(3) $$\begin{array}{l} \frac{d\left[Ca\right]}{dt}=\varepsilon \left(-\mu \;I_{Ca}-k_{Ca}\left[Ca\right]\right). \end{array}$$

*n* is a gating variable for the potassium current obeying:

(4) $$\begin{array}{l} \frac{dn}{dt}=\frac{\phi \left(n_{\infty }\left(v\right)-n\right)}{\tau_{n}\left(v\right)}, \end{array}$$

(5) $$\begin{array}{l} n_{\infty }\left(v\right)=0.5\left(1+\tanh\left(\frac{v-v_{3}}{v_{4}}\right)\right), \end{array}$$

(6) $$\begin{array}{l} \tau_{n}\left(v\right)=1/cosh\left(\frac{v-v_{3}}{2v_{4}}\right). \end{array}$$

Synaptic variable, *s*, is modeled by an equation for the fraction of activated channels:

(7) $$\begin{array}{l} \frac{ds}{dt}=\alpha s_{\infty }\left(v\right)\left(1-s\right)-\;\beta s, \end{array}$$

where $s_{\infty }\left(v\right)=1/\left(1+\exp\left(-\frac{v+\theta_{s}}{\sigma_{s}}\right)\right).$ The term *I*_*syn*_ in Equation (1) represents the synaptic input from the other M-cell and given by *I*_*syn*_ = *g*_*syn*_(*v* − *v*_*syn*_)*s*, where *s* is the synaptic variable from another M-cell.

The applied current *I*_*app*_(*t*) in the M-cell for *i* = 1, 2 is modeled as:

(8) $$\begin{array}{l} I_{app}\left(t\right)=I_{0}+I_{i}\left(\tau \right)+w_{M}\times E_{net}\left(t\right), \end{array}$$

where *I*_0_ is a fixed constant, *I*_*i*_(τ) is the stimulus at time τ , *w*_*M*_ is a fixed constant for the weight.

*E*_*net*_(*t*) represents an activity-dependent adaptation with respect to repeated sensory inputs. The M-cell receives both excitatory inputs from the VIII^th^ sensory nerve and inhibitory inputs from inhibitory commissural neurons (reviewed in Zottoli and Faber, 2000; also in Korn and Faber, 2005). Habituation is affected by a combination of increased inhibitory input from feed-forward inhibitory neurons and decreased excitatory input from auditory afferents, which decrease the net excitability of the M-cell (Marsden and Granato, 2015). We extend this idea so that in the model, *E*_*net*_(*t*) (Equation 9) represents the maximal net pre-synaptic input to the M-cell. Here we assume that *E*_*net*_(*t*) is excitatory. Now, calcium is known to modulate inhibitory pre-synaptic neurotransmitter release via retrograde signaling (Diana and Bregestovski, 2005). Pre-synaptic release of dopamine and post-synaptic activation of the M-cell are also known to be calcium dependent (Cachope et al., 2007). The M-cell's calcium response is due to the calcium influx to the M-cell (Takahashi et al., 2002) and reflects the M-cell's activation (Marsden and Granato, 2015). Moreover, the startle escape response probability of the M-cell is determined by the amplitude of calcium in the dendrite of the M-cell (Marsden and Granato, 2015). Hence, in the model M-cell we assumed that intracellular calcium level reciprocally modulates the maximal net excitation of the M-cell. Now, activity-dependent adaptation *E*_*net*_(*t*) obeys the following equation:

(9) $$\begin{array}{l} \frac{d\;E_{net}}{dt}=\frac{\left[\frac{ag_{max}}{{\left[Ca\right]}_{i}+k_{2}}\;-\;E_{net}\right]}{\rho }. \end{array}$$

where ag_max_ is the maximal net excitation, ρ is the time constant of *E*_*net*_(*t*), and [Ca]_*i*_ is the intracellular calcium concentration of the *i*-th M-cell.

The set of parameter values are given by the following unless specified otherwise. *g*_*Ca*_ = 4, *g*_*KCa*_ = 0.25, *g*_*K*_ = 8, *g*_*L*_ = 2, ε = 0.00033, *v*_*Ca*_ = 120, *v*_*K*_ = −84, *v*_*L*_ = −60, *k*_1_ = 10, *k*_2_ = 40, θ_*s*_ = 0, *v*_1_ = −1.2, *v*_2_ = 18, *v*_3_ = 12, *v*_4_ = 17, *k*_*Ca*_ = 1, *mu* = 0.2, *c*_*M*_ = 1, ϕ = 0.23, α = 10, β = 0.08, *g*_*M*→*M*_ = 0.5, *v*_*M*→*M*_ = −50, *s*_2_ = 0.029, δ_*s*_ = 4, *I*_*o*_ = 40.5, *w*_*M*_ = 0.5, *I*_1_(τ) = 4.5, *I*_2_(τ) = 0, *ρ* = 8400. Parameter values are adjusted in such a way that the M-cells do not fire action potentials unless they receive enough excitatory inputs from external stimuli.

In the computational study, we used the maximal net excitation ag_max_ (Equation 9) as the main parameter to explore how the change in neuromodulatory inputs to the M-cell circuit leads to differences in habituation rate to repeated stimuli. Large ag_max_ values presumably represent subordinate-like social phenotype, intermediate ag_max_ values communal-like phenotype, and small ag_max_ values dominant-like phenotype. **Figure 3** shows one possible schematic illustration of the model M-cell with excitatory and inhibitory inputs to the M-cell with large ag_max_ (**Figure 3A**), intermediate ag_max_ (**Figure 3B**), and small ag_max_ (**Figure 3C**).

To simulate the effect of an external stimulus onto the M-cell, a depolarizing current pulse was applied to the M-cell model neuron. Simulations were performed on a personal computer using the software XPP (Ermentrout, 2002). The numerical method used was an adaptive-step fourth order Runge-Kutta method with a step size 0.01 ms.

## Results

### Experimental results

**Figure 2:**
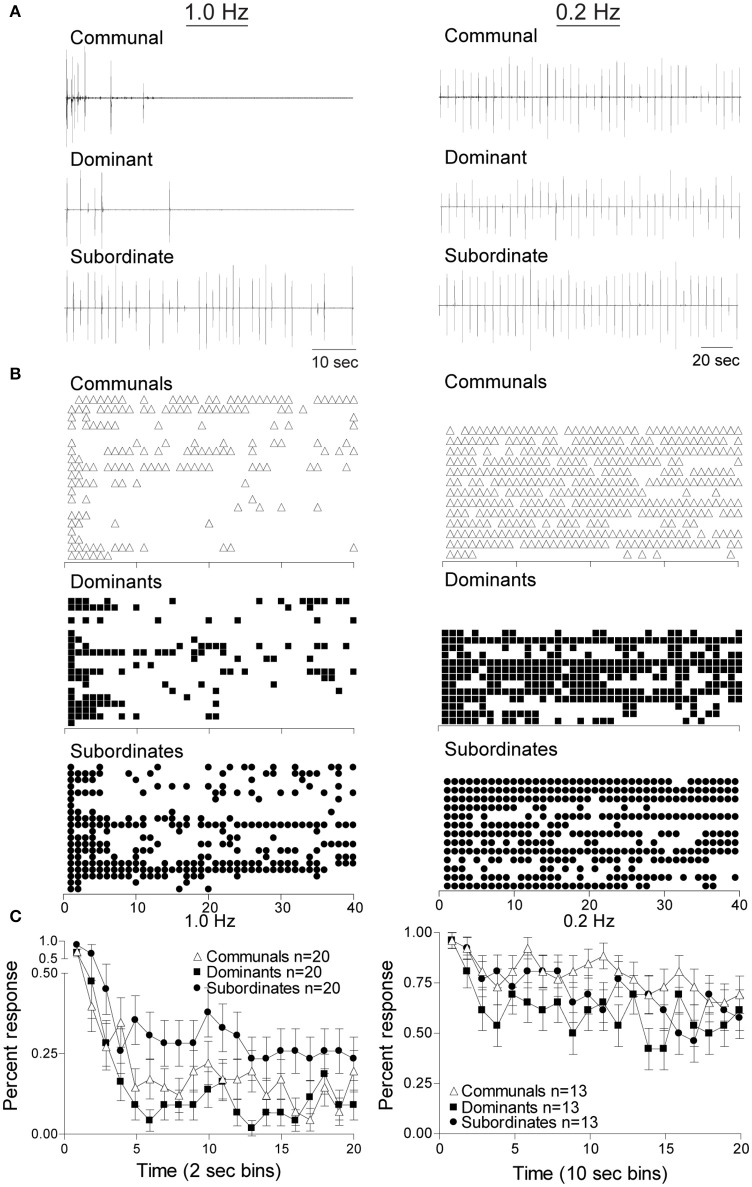
Social status affects the response rates of startle escape. Repeated auditory stimuli at 1 Hz (left panel) causes rapid habituation in all animal groups particularly in dominant animals. However, stimuli at 0.2 Hz (right panel) leads to modest habituation in all animals groups. **(A)** Individual example of response pattern of a communal animal and one pair of dominant and subordinate animals to repeated stimuli at 1 Hz (left panel) and 0.2 Hz (right panel). Deflections are M-cell mediated field potentials. **(B)** Raster plots of all animals tested. Each row represents the responses of one animal, and each symbol represents one M-cell mediated escape response. **(C)** Cumulative percent response patterns binned over 2 s periods (left panel) at 1 Hz and 10 s period (right panel) at 0.2 Hz for each group. Mean ±SEM was plotted.

To determine the effects of social status on habituation of the M-cell mediated escape response we delivered repetitive supra-threshold auditory stimuli (Figures 1B,D). Figure 2 shows the response patterns of dominants, subordinates, and communals to consecutive 40 stimuli at 1 Hz (left panel) and 0.2 Hz (right panel), respectively. Here, male communal animals of similar age and size were randomly selected from mixed sex tanks of ~20 fish. We found that 1 Hz auditory stimulation was effective in habituating the escape response in all three groups. The rates of habituation were modestly higher in dominants and lower in subordinates compared to communals at 1 Hz. However, the rate of habituation in dominants was much more pronounced compared to subordinates. With repeated stimuli at 1 Hz, subordinates continued to faithfully respond at a steady rate; however, most dominant and some communal animals stopped responding after the first few repeated stimuli (Figure 2 left panels). When the same experiment was repeated with a 0.2 Hz stimulation protocol, we found that the habituation rate was reduced and differences of habituation rates among the three animal groups became less pronounced (Figure 2 right panels).

To determine whether these observations were statistically significant among the three social phenotypes and stimulus frequencies, we conducted a mixed design ANOVA (within-subject factor as stimulation (40 stimuli), between-subject factors as group (dominant, subordinate, and communal animals) and frequency (1 and 0.2 Hz). We found that there were significant main effects of stimulation [*F*_(39, 3,627)_ = 1.04e+1, *p* < 1.0e-16], frequency [*F*_(1, 93)_ = 1.07e+2, *p* < 1.0e-16], and group [*F*_(2, 93)_ = 3.36, *p* = 3.88e-2]. There was also a two-way interaction between frequency and stimulation [*F*_(39, 3,627)_ = 2.34, *p* = 5.53e-6]. But there were neither two-way nor three-way interactions between frequency and group [*F*_(2, 93)_ = 1.76, *p* > 0.05], between group and stimulation [*F*_(78, 3,627)_ = 8.87e-1, *p* > 0.05], and among factors (group, frequency, and stimulation) [*F*_(78, 3,627)_ = 9.00e-1, *p* > 0.05].

We further performed *post-hoc* tests to determine the differences in the average response rates for animal groups and for the test conditions (1 and 0.2 Hz). The *post-hoc* test showed that the average response rates of the startle escape for subordinates were significantly higher compared to dominants (Tukey's HSD, *p* = 4.53e-2). However, there were no differences in the average response rates of the startle escape between dominants and communals (Tukey's HSD, *p* > 0.05) and between subordinates and communals (Tukey's HSD, *p* > 0.05). Moreover, the *post-hoc* test showed that the average response rates of the startle escape at 0.2 Hz were significantly higher compared to 1 Hz (Tukey's HSD, *p* = 1.06e-10).

Additionally, we analyzed differences of the response rates among animal groups at each frequency (1 and 0.2 Hz). To investigate the response rates of the startle escape in three animal groups (communal, dominant, and subordinate) at 1 Hz, a mixed-design ANOVA (within-subject factor as stimulation, between-subject factor as group) was performed. We found that there were significant main effects of stimulation [*F*_(39, 2,223)_ = 1.17e+1, *p* < 1.0e-16] and group [*F*_(2, 57)_ = 3.85, *p* = 2.71e-2]. But there was no effect of interaction between stimulation and group [*F*_(78, 2,223)_ = 8.77e-1, *p* > 0.05]. We further performed *post-hoc* test to determine the differences of the response rates between animal groups at 1 Hz. The *post-hoc* test indicated that the response rates of the startle escape of subordinates were significantly higher compared to dominants (Tukey's HSD, *p* = 2.54e-2), but no other differences were observed (Tukey's HSD, *p* > 0.05). Moreover, as illustrated in Figure 2C left panel we observed the response rates of the startle escape of the first few stimuli for all animal groups were at least twice higher compared to the rest of the stimuli. To explore this observation further, we pooled the time bin into every 5 stimuli (1–5, 6–10, 11–15, etc.) and then performed a mixed-design ANOVA (within-subject factor as stimulation, between-subject factor as group). There were significant main effects of stimulation [*F*_(7, 399)_ = 2.75e+1, *p* < 1.0e-16] and group [*F*_(2, 57)_ = 3.85, *p* = 2.71e-2]. No effect of interaction between stimulation and group was observed [*F*_(14, 399)_ = 5.29e-1, *p* > 0.05]. The *post-hoc* test indicated that the response rates of the startle escape of the first pooled time bin (1–5 stimuli) was significantly higher compared to all other time bins (6–10, 11–15, …, 35–40) (Tukey's HSD, *p* ≤ 6.80e-8). There were no other differences among all other time bins (Tukey's HSD, *p* > 0.05) for all animal groups. This indicates that the response rates of the startle escape at 1 Hz stimuli were quickly decreased within the first five stimuli. After the first five stimuli, the response rates were settled down to certain levels depending on animal groups.

We also investigated the response rates of the startle escape for all three animal groups at 0.2 Hz by using a mixed-design ANOVA (within-subject factor as stimulation, between-subject factor as group). There was a significant main effect of stimulation [*F*_(39, 1,404)_ = 3.12, *p* = 7.02e-10] but no effect of group [*F*_(2, 36)_ = 1.70, *p* > 0.05]. There was also no effect of interaction between stimulation and group [*F*_(79, 1,404)_ = 8.40e-1, *p* > 0.05]. As we did at 1 Hz, we also pooled the time bin into every 5 stimuli to determine whether the response rates of the startle escape of the first 5 stimuli were different from other time bins. We observed a significant main effect of stimulation [*F*_(7, 252)_ = 5.97, *p* = 1.92e-6] at 0.2 Hz although it was lower compared to the effect observed in the 1 Hz protocol. There were no main effect of group [*F*_(2, 36)_ = 1.70, *p* > 0.05] and no two-way interaction between stimulation and group [*F*_(14, 252)_ = 8.48e-1, *p* > 0.05]. *Post-hoc* test indicated that the response rate of the startle escape of the first pooled time bin (1–5 stimuli) was significantly higher compared to all other time bins (Tukey's HSD, *p* ≤ 3.28e-2) except the third pooled time bin (11–15 stimuli) (Tukey's HSD, *p* > 0.05). There were no other differences among all other time bins (Tukey's HSD, *p* > 0.05). This indicates that the response rates of the startle escape to the 0.2 Hz repeated stimulation were also decreased within the first 5 stimuli although rates of decrease were smaller compared to 1 Hz. After the first 5 stimuli, the response rates were settled down to certain levels depending on animal groups.

In summary, our results show that the habituation to repeated stimulation occurred at both frequencies (1 and 0.2 Hz) for all animal groups. However, at the moderate frequency (1 Hz) the habituation rates were more prominent compared to the lower frequency (0.2 Hz). The habituation rates for dominants were significantly higher compared to subordinates at 1 Hz while the differences of the habituation rates among all three animal groups were disappeared at 0.2 Hz. These results suggest that both social experience and frequency of stimulus influence the habituation of the M-cell's escape circuit.

### Numerical results

#### Activity patterns and irregularity

**Figure 3:**
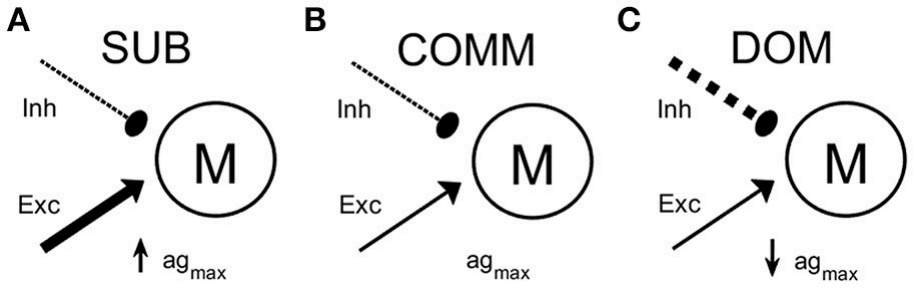
A schematic illustrations of the M-cell escape circuit for all animal groups. The M-cell receives excitatory input (solid line with arrow head) and inhibitory input (dashed line with filled circle). **(A)** Subordinate-like case (SUB) with higher ag_max_. **(B)** Communal-like case (COMM) with moderate ag_max_. **(C)** Dominant-like case (DOM) with lower ag_max_. Thickness represents the strength of the input.

Excitability of the startle escape circuit is subject to descending neuromodulation (Oda et al., 1998; Preuss and Faber, 2003). Pre-synaptic inputs to the M-cell may modulate the excitability of the startle escape circuit by changing in the excitability of the M-cell (Cachope et al., 2007; Medan and Preuss, 2011; Whitaker et al., 2011; Marsden and Granato, 2015). However, little is known of how a change in the M-cell's excitability results in different startle responses depending on social status and the properties of the stimulation input. Here, we hypothesized that pre-synaptic inputs to the M-cell escape circuit may be changed differentially according to social status and characteristics of the stimulations, which accounts for the observed differences in the response rates between dominants and subordinates and in the frequencies of the stimulation inputs. To test this hypothesis, we used the neurocomputational model of the M-cell (Miller et al., 2017; Equations 1–9) as a simplified M-cell escape circuit. This M-cell model is based on a conductance-based modified Morris-Lecar neuronal model (Morris and Lecar, 1981; Izhikevich, 2007; Ermentrout and Terman, 2010) with additional calcium-dependent potassium current. In the simulation of the model, the maximal net excitation (ag_max_) in the M-cell, which reflects the total net pre-synaptic inputs to the M-cell, was used as a main control parameter. Here, we assumed that the social status differentially affect the total pre-synaptic inputs to the M-cell escape circuit (see Materials and Methods). For example, the subordinate-like model receives more total net excitatory inputs so that it has higher ag_max_ (Figure 3A) while the dominant-like model receives less total net excitatory inputs so that it has lower ag_max_ (Figure 3C). The communal-like model lies in between the dominant-like model and the subordinate-like model (Figure 3B). In the computational study, we explored not only the effects of social status, but also the effects of the characteristics of the stimulus input including the magnitude and the frequency.

**Figure 4:**
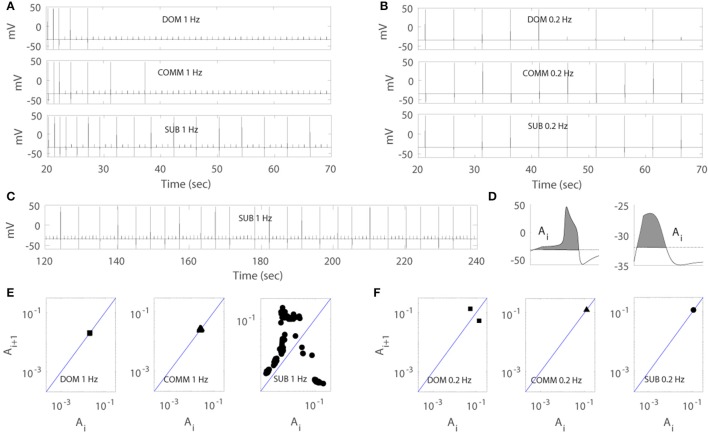
Numerical simulation of the model for dominant-like case (DOM) (ag_max_ = 41.5 upper panels), communal-like case (COMM) (ag_max_ = 42.2 middle panels), and subordinate-like case (SUB) (ag_max_ = 43.5 lower panels) under periodic inputs at 1 Hz (left panels) and 0.2 Hz (right panels). Membrane voltage trace in DOM (upper panel), COMM (middle panel), and SUB (lower panel) at 1 Hz **(A)** and 0.2 Hz **(B)**. (**C**) Irregular activity patterns of SUB at 1 Hz (corresponds to E right panel). **(D)** Area *A*_*i*_ under the voltage trace above the threshold (dash line) with action potential (left panel) and subthreshold (right panel). **(E,F)** One-dimensional return map in DOM (left panel), COMM (middle panel), and SUB (right panel) at 1 Hz **(E)** and 0.2 Hz **(F)**.

Figures 4A,B illustrated that the model was able to generate different response patterns depending on different maximal net excitation values (ag_max_). The simulation began by finding quasi-steady states of membrane voltage *v*, gating variable *n*, calcium concentration [Ca], activity-dependent adaptation *E*_*net*_ by running the computer simulation for 20 s without any stimulus input. Periodic depolarizing current pulses at 1 Hz (left panels) or 0.2 Hz (right panels) were given with the above initial conditions at time *t* = 20.3 s. For ag_max_ = 41.5, the model displayed dominant-like response phenotype whereby the model faithfully responded only to the first few stimuli (Figure 4A upper panels). For ag_max_ = 42.2 the model displayed communal-like response phenotype similar to the experimental results illustrated in Figure 2 (Figures 4A,B middle panels). On the other hand, for ag_max_ = 43.5 the model displayed subordinate-like response phenotype similar to the experimental results illustrated in Figure 2 (Figures 4A,B lower panels). As ag_max_ increased, the neuronal model tended to respond more faithfully to repeated stimulation inputs and eventually showed full and faithful responses to the stimulus inputs. In summary, the model was able to reproduce different degrees of habituation under periodic stimulation at 1 and 0.2 Hz that mimicked the experimental results shown in Figure 2 by controlling the parameter ag_max_. In the following analysis, we further explored how social experience and characteristics of the repeated stimulations affect habituation process.

Due to the important role of excitable cells in information processing within neural networks and many other biological systems, the response of excitable cells under periodic input has been studied extensively. For example, Kaplan et al. (1996) studied the response of periodically stimulated squid giant axons and observed irregular action potentials. Using one-dimensional return maps, they found that deterministic subthreshold dynamics are responsible for the observed irregular response patterns and subthreshold responses modulate the response of neurons to subsequent stimuli. In fact, the M-cell in our model showed similar response patterns to periodic input. As the simulation persisted, the cell tended to skip the response to inputs and elicited less action potentials and eventually showed irregular response patterns (Figure 4C). To unveil the deterministic subthreshold dynamics responsible for this irregularity in our model, we constructed one-dimensional map using 200 s long data (between 100 and 300 s after the initiation of stimulation to obtain stable responses). Following Kaplan et al. (1996), we used the areas under the voltage trace around each stimulation input because the shape of action potentials and subthreshold responses reflect the dynamics of the neuron under repeated stimulation. More precisely, we first set up a certain threshold and then computed the area (denoted by *A*_*i*_) under the voltage trace above this threshold at each stimulation *i* (Figure 4D). We then plotted (*A*_*i*_, *A*_*i*+1_). As in Kaplan et al. (1996), a logarithmic scale was used because of the large difference between the area of an action potential and a subthreshold response. Figures 4E,F showed the resulting one-dimensional maps at 1 Hz (Figure 4E) and 0.2 Hz (Figure 4F). Note that the maps do not depend strongly on how the areas are calculated as stated in Kaplan et al. (1996). The solid line on the diagonal in each figure is the line of identity where *A*_*i*+1_ = *A*_*i*_. The small cluster of points on the line of identity in Figure 4E left and middle panels implied that the subthreshold responses of dominant-like and communal-like under 1 Hz stimulation were regular. On the other hand, the Λ –shape of the map in Figure 4E right panel (subordinate-like) suggested that the observed irregularity was governed by a deterministic one-dimensional map (see Figure 4C for the irregularity of the voltage trace). In fact, as the social-status dependent parameter, ag_max_, increases, the return maps can be categorized into 5 different types. The first one was a map with a stable subthreshold fixed point as shown in Figure 4E left and middle panels, where the cell eventually stops responding to repeated inputs (dominant-like and communal-like at 1 Hz). A map with a stable subthreshold periodic orbit was the second type, where the cell elicits sparse but periodic action potentials (few clusters of points) where at least one cluster was on the identity line and others were away from the identity line (between Figure 4E middle panel and Figure 4E right panel). The third type was a subthreshold irregular (potentially chaotic) pattern as shown in Figure 4E right panel. The fourth was a supra-threshold periodic orbit, where the action potentials were skipped sparsely but periodically (Figure 4F left panel). The fifth was a map with a supra-threshold fixed point, where the cell fired faithfully to the repeated inputs (subordinate-like and communal-like in Figure 4F middle and right panels).

#### Modulation of response patterns by [Ca] and *E*_*net*_

A previous study has shown that the change in M-cell excitability is responsible for the change in startle plasticity (Neumeister et al., 2010). On the other hand, intracellular calcium is known to modulate the pre-synaptic inhibitory synaptic transmission via retrograde signaling, which is called depolarization-induced suppression of inhibition (Diana and Bregestovski, 2005); depolarization results in the increase of intracellular calcium concentration through voltage sensitive calcium channels, which is followed by retrograde transmitter release. Hence, we focused on the dynamics of [Ca] and activity-dependent adaptation *E*_*net*_ in response to repeated stimulation inputs to study how the cooperation of two slow variables [Ca] and *E*_*net*_ modulates the response of the M-cell that will lead to different response patterns depending on ag_max_. More precisely, in this section, we explored (1) how the habituation of the M-cell escape response to 1 Hz periodic inputs depends on [Ca] and *E*_*net*_ and (2) how the irregular activity patterns in subordinates are obtained.

**Figure 5:**
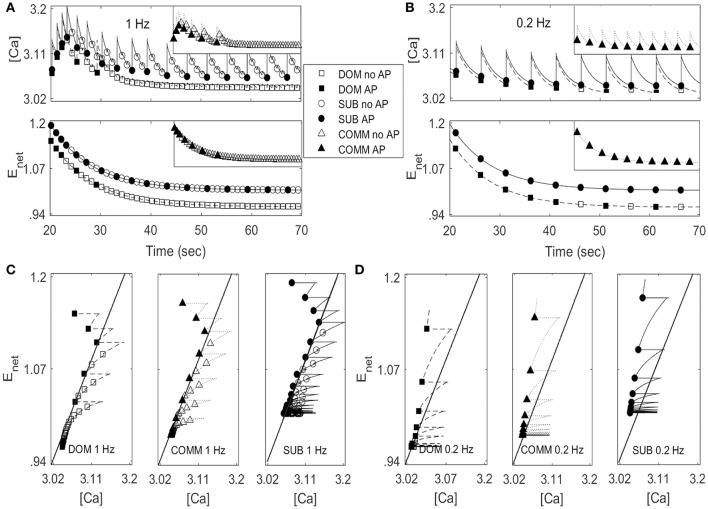
Activity patterns of [Ca] and *E*_*net*_ in dominant-like case (DOM) (lower curves with square symbols) and subordinate-like case (SUB) (upper curves with circle symbols) under periodic inputs at 1 Hz **(A)** and 0.2 Hz **(B)**. Insect in each panel in **(A,B)** show activity patterns of [Ca] and *E*_*net*_ in communal-like case (COMM) (triangle symbols) in a half-size scale. These activity patterns lie in between DOM and SUB. Circle, triangle, and square symbols denote the moments when inputs are given. Closed symbols denote that the cell fires action potentials (AP) accordingly while open symbols denote no action potential (no AP). **(C,D)** ([Ca], *E*_*net*_)-space with the projection of the solution trajectory in DOM (left panel), COMM (middle panel), and in SUB (right panel) at 1 Hz **(C)** and 0.2 Hz **(D)**. The solid straight line that cuts the figure from lower left to upper right is the jump-up curve.

**Figure 6:**
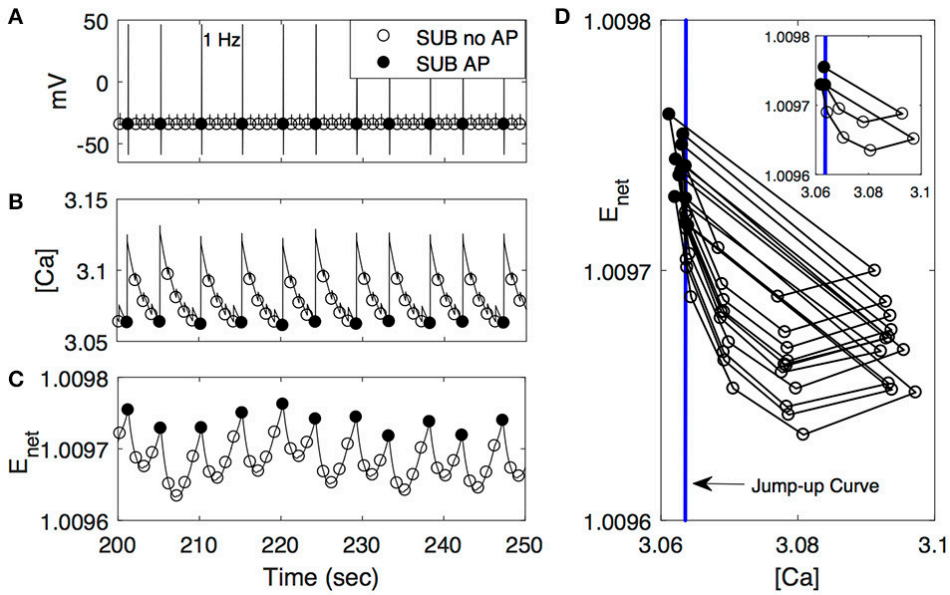
Activity patterns of voltage **(A)**, [Ca] **(B)**, and *E*_*net*_ **(C)** in subordinate-like case (SUB) from 200 to 250 s at 1 Hz. As in Figure 5, closed circle denotes that the cell fires action potentials (AP) accordingly while open circle denotes no action potential (no AP). **(D)** ([Ca], *E*_*net*_)-space with the simplified projection of the solution trajectory with the jump-up curve (vertical line). The insert figure plots only the first 10 solution trajectories.

With the same data sets in Figure 4 for dominant-like, communal-like, and subordinate-like cases, we plotted the temporal profiles of [Ca] and *E*_*net*_ in Figures 5A,B. The lower curve in each figure corresponded to dominant-like (square symbols) and the upper curve for subordinate-like cases (circle symbols). The inset in each figure showed the temporal profile for communal-like case (triangle symbols) in a half-size scale. Closed symbols in each figure denoted the moments when action potentials were elicited by stimulation while open symbols denoted the moments with no action potentials. When the input was given, the cell was depolarized and [Ca] increased rapidly. On the other hand, when the stimulation was terminated [Ca] decreased slowly. *E*_*net*_ also decreased slowly to periodic inputs although *E*_*net*_ demonstrated more subtle dynamics which we will investigate in more detail later in Figure 6. The overall levels of both [Ca] and *E*_*net*_ decreased over the repeated stimulations. To explore how the cooperation of two slow variables [Ca] and *E*_*net*_ modulate the response of the cell to periodic stimulations, we considered two-dimensional ([Ca], *E*_*net*_)-space in more detail to get better insights of the roles of these two slow variables.

Figures 5C,D show ([Ca], *E*_*net*_)-space with the projection of dominant-like case (left panel), communal-like case (middle panel), and subordinate-like case (right panel) at 1 and 0.2 Hz stimulus inputs. The trajectory in each case started at the upper part of the figure and moved downward. The same symbols in Figures 5A,B were also used in Figures 5C,D. The line that runs through from the lower left corner to the upper right corner in each figure was a jump-up curve, which was numerically computed as follows. Figures 5A,B showed that [Ca] lied in [3, 3.2] and *E*_*net*_ in [0.9, 1.2] when periodic inputs were given. Therefore, we focused on the rectangular region, R = [3, 3.2] × [0.9, 1.2] in the slow ([Ca], *E*_*net*_)-space and chose grid points with increment 0.01 in horizontal and vertical directions. Since the membrane voltage *v* and gating variable *n* were fast variables, we assumed that *v* and *n* approached their steady states quickly. When stimulation was given, we assumed that *v* = −34.32 and *n* = 0.00427 in dominant-like case and communal-like case, and *v* = −34.322 and *n* = 0.00429 in subordinate-like case. Note that the initial values of the membrane voltage *v* and gating variable *n* for dominants and subordinates were slightly different because their parameter values ag_max_ were different. After the finite time simulation, their steady state values were also different. For each grid point in the rectangular region R, we used the values of [Ca] and *E*_*net*_ along with the fixed values of *v* and *n* as initial conditions and determined whether the cell fired when the stimulation was delivered at the time *t* = 20 s. In Figures 5C,D, the region left to the curve was a jump-up region where the cell fired whenever the simulation input was given.

In all three (dominant-like, communal-like, and subordinate-like) cases, the trajectory began at the jump-up region, which explains why the cell showed faithful responses to the first few periodic stimulation inputs. However, for the dominant-like and communal-like cases at 1 Hz, the trajectory quickly escaped the jump-up region and remained outside the jump-up region. These response patterns of the three models can be explained by the dynamics of [Ca] and activity-dependent adaptation *E*_*net*_. Note that the overall level of [Ca] initially increases but eventually decreases and levels off while the overall level of *E*_*net*_ monotonically decreases and eventually levels off (Figures 5A,B). The incorporation of these two overall behaviors results in a reversed C-shaped trajectory in ([Ca], *E*_*net*_)-space. Therefore, the initial increase of overall [Ca] level along with monotonic decrease of overall *E*_*net*_ level opens up a possibility of escaping from the jump-up region. In addition, ag_max_ determines the steady-state value of *E*_*net*_ under no stimulation; specifically, bigger ag_max_ means bigger steady-state *E*_*net*_ value under no external stimulation inputs. Hence, ag_max_ determines how long the cell responds to the periodic inputs. For example, if ag_max_ is sufficiently large then the trajectory tends to stay within the jump-up region; hence, the cell elicits action potentials for each input. For intermediate values of ag_max_, the cell stays within the jump-up region for a while and either escapes (dominant-like case) or stays near the jump-up curve to generate intermittent responses (subordinate-like case). If ag_max_ is sufficiently small, then the trajectory starts outside the jump-up region; hence, the cell does not elicit any action potential. In summary, the incorporation of [Ca] and *E*_*net*_ dynamics is responsible for the escape from the jump-up region while ag_max_ (the maximal net excitation) modulates the duration of the stay within the jump-up region.

We further explored how calcium and net excitability determine the irregular escape response of the M-cell to repeated stimulations shown in the subordinate-like case. Figure 6 illustrates the profiles of membrane voltage *v*, calcium concentration [Ca], and activity-dependent adaptation *E*_*net*_ from 200 to 250 s since the initiation of stimulation. Apparently membrane voltage *v* shows sparse and irregular action potentials. Intracellular calcium concentration [Ca] increases rapidly whenever the cell receives an external input, hence whenever the cell is depolarized. This increment is very large if the cell elicits an action potential and small if not. Between depolarization events driven by a series of external inputs, either an action potential or subthreshold depolarization, [Ca] decreases slowly. On the other hand, the dynamics of *E*_*net*_ is slower than [Ca] and the overall behavior of *E*_*net*_ between action potentials is not monotonic, which is an important substrate for the generation of irregular response to the external inputs. Whenever the cell fires an action potential, *E*_*net*_ decreases initially and then begins to increase until the next action potential since the dynamics of *E*_*net*_ is regulated by [Ca] (Equation 9). Now, when the cell fires an action potential, the trajectory in ([Ca], *E*_*net*_)-space escaped the jump-up region rapidly. During subsequent inputs, the cell is unable to generate action potentials since the decrement of [Ca] is not sufficient to push back the trajectory into the jump-up region. However, the overall [Ca] decreases slowly and *E*_*net*_ increases slowly, hence the trajectory is eventually pushed back into the jump-up region. For this re-injection mechanism, the baseline level of [Ca] under repeated inputs should be sufficiently small to ensure that the trajectory is pushed back into the jump-up region and sufficiently close to the jump-up curve to ensure the occurrence of irregular response. Considering that ag_max_, the maximal net excitation, determines the baseline level of *E*_*net*_ under repeated stimulation inputs, irregular responses can happen for some values of ag_max_ as shown in numerical simulation. The dynamics of *E*_*net*_, which decreases initially and then slowly increases, also modulates the occurrence of irregular action potentials.

Figure 6D illustrates that the condition of the trajectory within the jump-up region does not fully guarantee the cell would fire an action potential. In some cases, the cell is unable to fire an action potential even though the trajectory is within the jump-up region and vice versa. Recall that when we constructed the jump-up region, we assumed that membrane voltage *v* and gating variable *n* would approach their steady-state values quickly. We chose quasi-steady state values for *v* and *n* and used them as initial conditions for *v* and *n* throughout the numerical simulation to determine the jump-up region. When the trajectory is away from the jump-up curve but within the jump-up region, the constructed jump-up curve provides a good explanation of the response patterns of the cell to the inputs. However, when the trajectory is sufficiently close to the jump-up curve, the values of *v* and *n* at the time of the stimulation became critical in the generation of an action potential.

#### Response rates of cell to repeated stimuli

In this section, we explored how the response rates of the model M-cell to a series of repeated inputs change when ag_max_ and particular characteristics (frequency and amplitude) of the repeated stimuli were varied. More precisely, we measured the Faithfulness of the response of the cell to the external stimulations by changing ag_max_ and stimulus frequency and amplitude. Here, the Faithfulness of the response of the cell to repeated stimuli is defined as the following:

(10) $$\begin{array}{l} Faithfulness=\frac{the\;number\;of\;the\;response\;of\;cell}{the\;number\;of\;repeated\;stimulations} \end{array}$$

This value varies from 0 (no response) to 1 (full response). We measured this value during two different time intervals: initial time interval (20–30 s) and stable time interval (40–70 s). Note that the stimuli began at time 20 s to ensure that the cell was at the quasi-steady state. By measuring the Faithfulness of the cell to the repeated stimuli for two different time intervals, we explored how ag_max_ and characteristics of the stimulation inputs affect the response rates of the cell to the repeated stimuli. That is, we used our neurocomputational model to explore the response rates of the M-cell on various conditions beyond the empirical study. For example, we changed the amplitudes and frequencies of the repeated stimulations along with various levels of ag_max_, which mimics the difference of the social status in the empirical study. We also considered two different time scales to compare how the Faithfulness occurs in different conditions. Note that higher Faithfulness corresponds to lower habituation rate while lower Faithfulness corresponds to higher habituation rate.

**Figure 7:**
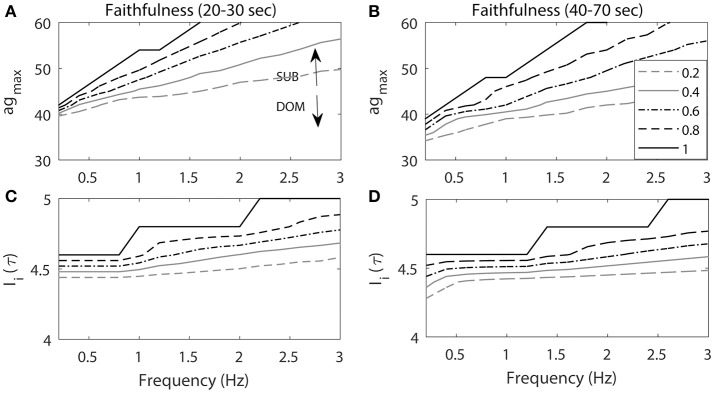
The projection of contour level curves of Faithfulness onto the plane. Faithfulness of M-cell with the frequency and ag_max_ **(A,B)** and amplitude of the stimulation input *I*_*i*_(τ) **(C,D)**. **(A,C)** Faithfulness during 20–30 s period when the repeated stimulations delivered at 20 s. (**B**, **D**) Faithfulness during 40–70 s period. High ag_max_ resembles the activity patterns for subordinate-like case (SUB) while low ag_max_ resembles the activity patterns for dominant-like case (DOM). Different curves represent different values of Faithfulness.

Figure 7 illustrates that the Faithfulness of the cell to repeated stimuli depended on both ag_max_ and the characteristics of the repeated stimuli including the frequency and the amplitude of the stimulation input. Figures 7A,B shows the Faithfulness when the frequency (Hz) and ag_max_ were changing. As ag_max_ increased, Faithfulness also increased. Note that *E*_*net*_ is the positive excitatory input to the cell and is an increasing function of ag_max_. Thus, it is easier for the cell to respond to the stimulation input with higher ag_max_ and *E*_*net*_, which results in higher Faithfulness. This implied that the subordinate-like case (higher ag_max_) had higher values of Faithfulness compared to the dominant-like case (lower ag_max_).

On the other hand, as the frequency increased, *E*_*net*_ could not be fully recovered from the previous activity due to the previous external stimulation input and this led to lower Faithfulness values. Note that when the frequency was small (<1 Hz), the different level curves converged. This implied that when the frequency was small (longer inter stimulus interval), the cell had enough time to recover from the previous stimulation input so that it could faithfully respond to the next stimulation. Since *E*_*net*_ decreased as the cell fired, the response rates of the cell also decreased as well. Thus, we observed that Faithfulness during the 40–70 s period (Figure 7B) was lower compared to the first 20–30 s period (Figure 7A). This indicated that the cell exhibited lower response rates of escape to the repeated stimulation inputs as a function of time.

Figures 7C,D illustrates Faithfulness when the stimulus frequency and the magnitude were changed. As the stimulus magnitude increased, it was easier for the cell to respond to the stimulation, which consequently led to higher values of Faithfulness. Miller and colleagues showed that the response probability of the M-cell to repeated stimuli is an increasing function of the amplitude of the input (Miller et al., 2017). The effect of the frequency was the same as the one in the previous result. That is, the smaller the frequency, the higher the Faithfulness value. We also observed higher values of Faithfulness during the first 20–30 s period (Figure 7C), followed by lower values during the 40–70 s period (Figure 7D), which indicated that the cell might exhibit high habituation rates to the repeated stimuli.

## Discussion

In the present study, we investigated one possible mechanism of how social status affects habituation processes in zebrafish to repeated auditory stimulation. Three distinct social phenotypes (dominants, subordinates, and communals) were considered. To investigate a potential mechanism underlying the different habituation processes observed in these three groups, we used both empirical and neurocomputational model of the M-cell. Empirically, the M-cell habituated to repeated moderate frequency auditory stimulation and habituation rate of the M-cell was socially regulated: dominant animals habituated more readily compared to subordinates at 1 Hz (Figure 2). In fact, at this stimulus frequency, dominant animals habituated slightly quicker compared to communals while subordinate animals habituated slightly slower compared to communals. A decrease in stimulus frequency eliminated social status dependent differences in habituation rates, albeit habituation occurred at a much-reduced degree. Our computational study demonstrated that the total net excitability of the M-cell escape circuit played a crucial role in the reproduction of the different habituation processes observed empirically and suggested that social status may affect pre-synaptic inputs to the M-cell to result in different habituation processes observed experimentally. Our computational study also predicts that habituation was more pronounced as either the frequency of repeated stimulation increased or the intensity of the stimulus decreased (Rankin et al., 2009; Marsden and Granato, 2015).

Our empirical results showed that the habituation rates at 0.2 Hz were less prominent compared to the higher frequency (1 Hz) unlike Marsden and Granato (2015) that showed prominent habituation rates at 0.2 Hz. This conflicting results come probably from the difference in experimental conditions: Marsden and Granato (2015) applied acoustic-vibrational stimuli to 5 dpf zebrafish head-restrained larvae in a simple learning task with 30 stimuli of 13 dB (the first 5 stimuli at 1/120 Hz and the final 25 stimuli at 0.2 Hz) while we applied supra-threshold auditory stimuli of 95 dB with 40 stimuli to adult male zebrafish.

Descending serotonergic and dopaminergic modulatory inputs regulate the excitability of the escape circuit (Whitaker et al., 2011; Mu et al., 2012; Medan and Preuss, 2014; Pantoja et al., 2016). Serotonin and dopamine induce opposite effects on the excitability of the escape circuit in that their application can enhance or depress the activation of the M-cell. For example, reduction of serotonin increases the habituation while dopamine leads to an opposite effect (Pantoja et al., 2016). This neuromodulatory control is mediated via direct modulation of the pre-synaptic sensory and postsynaptic M-cell and indirectly by regulating the feed-forward and feed-backward inhibitory inputs that finely tunes excitability of the M-cell (Marsden and Granato, 2015). The collective excitatory and inhibitory inputs that influence the escape circuit can be modeled as changes in calcium dynamics represented by the activity-dependent adaptation *E*_*net*_, which is regulated by the maximal net excitation (ag_max_). In fact, calcium is known to control both pre-synaptic release of dopamine and post-synaptic activation of the M-cell (Cachope et al., 2007). Thus, we hypothesized that social status may affect the summative neuromodulatory inputs to the M-cell escape circuit, which, combined with intracellular calcium dynamics, results in a social status-dependent habituation of the escape circuit. In our model we assumed that decreased inhibitory drive and/or increased excitatory drive onto the M-cell escape circuit result in higher net excitatory inputs. This would correspond with the higher net excitability in subordinate-like social phenotype (Figure 3A). Similarly, increased inhibitory drive and/or decreased excitatory drive to the M-cell escape circuit results in the lower net excitatory inputs that corresponds to the lower net excitability in dominant-like phenotype (Figure 3C).

To test this idea, we simulated and analyzed the response of a simplified model of the M-cell with respect to repeated stimuli. Although the model did not include all the dynamics and the contributions of many neurotransmitters (2-AG, dopamine, serotonin, etc.) and pre-synaptic cells (excitatory and inhibitory) that may act *in vivo*, the model was able to reproduce important characteristics of habituation processes observed in animals of different social standing by controlling the maximal net excitation ag_max_. More precisely, different habituation processes were obtained by the incorporation of intracellular cellular calcium concentration [Ca] and activity-dependent adaptation *E*_*net*_, which is modulated by the maximal net excitation ag_max_. In other words, the dynamics of slow variables (quantities that change slowly over time) modulate the overall activity patterns of the whole system. As explained in Figure 5, the slow dynamics of [Ca] and *E*_*net*_ govern the response of the M-cell to repeated stimulation that leads to different responses depending on ag_max_ and the stimulation frequency. This could explain why the habituation of the M-cell occurs quickly in dominants compared to subordinates when the frequency of the periodic input is moderate (around 1 Hz). This also could explain why both animals respond only to the first few stimuli when the frequency is sufficiently high. In our model calcium concentration [Ca] regulates the calcium dependent potassium channel (*I*_*KCa*_) which is known to control neuronal excitability and spike frequency adaption (Vergara et al., 1998). The calcium dependent potassium channel is present in the peripheral nervous system and sensory system of zebrafish including the statoacoustic (VIII) ganglia (Cabo et al., 2013). Moreover, the existence of this channel in the M-cell of zebrafish was also suggested (Brewster, 2012). Further studies are necessary to determine the presence of the channel in M-cell of zebrafish.

The model also showed that the startle escape response rate is an increasing function of the frequency of the repeated input (Figure 7; Rankin et al., 2009). When the frequency was sufficiently low, all three (dominant-like, communal-like, and subordinate-like) models faithfully responded to the stimuli so that there was almost no habituation. This is because the M-cell had enough time to recover from the previous stimulation as the frequency decreases or the inter stimulation interval increases. In other words, *E*_*net*_ is able to increase sufficiently high while calcium concentration decreased sufficiently low, hence the M-cell lies either within or near the jump-up region where it was ready to fire an action potential when the next stimulation was delivered. On the other hand, when the frequency was sufficiently high, all three models (dominant-like, communal-like, and subordinate-like) stopped responding to the stimuli except the first few. This was because the *E*_*net*_ has no time to recover from the previous activity. This suggests that when the frequency is either too low or too high, the difference of the habituation rates among three models would disappear.

Within neuronal networks and many other biological systems, the response of excitable cells with respect to periodic input has been studied extensively due to its prevalence and importance in information processing (Ermentrout, 1996; Izhikevich, 2007; Smeal et al., 2010). Various activity patterns including silent, bursting, tonic spiking, and chaotic behaviors have been observed. The mechanisms underlying these activity patterns and transitions between them have been studied to obtain an insight into spatiotemporal patterns observed in neuronal networks (Prescott et al., 2008; Bogaard et al., 2009; Drion et al., 2015). Bifurcation theory in non-linear dynamics has been used to study neuronal excitability in depth (Ermentrout, 1996; Rinzel and Ermentrout, 1998; Izhikevich, 2000, 2007). The study of irregular patterns is a long lasting theme in non-linear dynamical systems and even in biological areas. For example, Kaplan et al. (1996) observed that the squid giant axon exhibits irregular action potentials under periodic inputs and found that deterministic subthreshold response dynamics underlies the observed irregular response. The model M-cell in the current study also exhibited irregular action potentials in subordinate-like cases; as the simulation persisted, the cell tended to skip the response to inputs and elicited less action potentials and eventually showed irregular response patterns. The corresponding one-dimensional return map showed that the underlying dynamics was deterministic (Figure 4). Figures 5, 6 illustrate that the dynamics of [Ca] and *E*_*net*_ determine the dynamics underlying the irregular response. The key substrate was the proximity to the jump up region when stimulation was given, which was in turn modulated by the maximal net excitation ag_max_. This was the reason why we obtained irregular response over a certain range of ag_max_ values.

The limitations of the current study and proposed model are noteworthy. First, this research is based on the experiment of only two different frequencies (1 and 0.2 Hz) and fixed stimulus intensity. To overcome these experimental limitations, we used a neurocomputational model and explored a wide range of the frequency and amplitude domains. Our computational modeling study replicated the empirical results and demonstrated that the rates of habituation of the M-cell startle escape response depend on the social status of the animals and characteristics (frequency and amplitude) of the stimulus inputs. Second, the usage of adult and freely behaving animals to measure habituation rates prevented the direct measurement of physiological changes in intracellular calcium of the M-cell during the habituation process. Although calcium imaging approaches are widely used in zebrafish research such as transgenic fish lines that express calcium indicators specifically in the M-cell, these studies are mostly limited to embryonic stages during which the skin is transparent and the skull is not yet fully formed. Thus, currently it is not possible to measure calcium dynamics in the M-cell of adult fish *in vivo*. Third, while the habituation rates were different depending on social conditions, the experimental results show that there were wide variations in the responsiveness of the M-cell to repeated stimuli within the same animal group. These individual differences may be due to the different activity levels of dorsal raphe nucleus serotonergic neurons (Pantoja et al., 2016). Pontoja and colleagues also showed that both serotonin and dopamine modulate the habituation in opposite directions. Moreover, 2-AG also modulates swimming and escape response in zebrafish (Song et al., 2015). Thus, it will be interesting to explore the effects of neuromodulators (including 2-AG, dopamine, serotonin, etc.) on the social status and the habituation of the M-cell. Fourth, we used a simplified single compartment computational model of the M-cell. One can expand this model by including additional pre-synaptic cells such as excitatory cells (like sensory VIII^th^ nerve fiber) and inhibitory cells (like commissural neurons) (reviewed in Zottoli and Faber, 2000; also in Korn and Faber, 2005). Moreover, the empirical results from calcium dynamics of the pre-synaptic cells and the M-cell during the escape response along with the changes of the neuromodulators will also help in building more elaborate computational models.

In conclusion, using a dual empirical and computational approach, we provided one possible mechanism of how social status affects habituation processes of the startle escape response in zebrafish. Social status may affect neuromodulatory inputs to the M-cell escape circuit that leads to differences in the M-cell's excitability. This may enable animals to readily learn to adapt to changes in their social environment by selecting the most appropriate behavior (for example, escape for subordinate animals while quick habituation or swim for dominant animals) to environmental stimuli. Our model suggests that the incorporation of calcium concentration [Ca] and activity-dependent adaptation *E*_*net*_ under the modulation of the maximal net excitation ag_max_ plays a critical role in reproducing different degrees of habituation rates depending on the social status and the periodic stimulation inputs. The change of the excitability of the M-cell may be due to the availability of 2-AG, hormonal regulation, or other neurotransmitters including dopamine and serotonin (Song et al., 2015; Pantoja et al., 2016). These mechanisms are not necessarily mutually exclusive. It is most likely that the synergistic action of few of these mechanisms may act in a cooperative manner. Current technological limitations prevent the direct exploration of the parameter space that regulates escape circuit dynamics. Therefore, it will be exciting when future technologies make it possible to empirically verify the proposed model by direct physiological measurements of the neuromodulatory inputs that regulate M-cell's excitability in socially experienced animals.

## Author note

Most animals exhibit habituation to repeated stimuli. However, how social experience and characteristics of the external stimulations affect habituation process is still poorly understood. Using zebrafish as an experimental model system, we showed that habituation is affected by social experience and is contingent on rate of stimulation. Dominant animals habituate rapidly compared to subordinates to repeated stimulation at a moderate frequency. However, as the stimulus frequency decreases, the difference of habituation rates between the two groups disappears. Moreover, the habituation rates of both social phenotypes at moderate stimulus frequency were higher compared to the low stimulus frequency. To test the idea that a change in neuromodulatory inputs to the M-cell may be responsible for the different habituation processes, we constructed a simplified computational model of the M-cell escape circuit. Our model showed that a change in total net excitability of the model M-cell escape circuit, which represents a summative neuromodulatory input to the M-cell, combined with intracellular calcium dynamics was sufficient to reproduce the experimental results. The model that represented “dominant-like” phenotype displayed rapid habituation compared to a model that represented “subordinate-like” phenotype at a moderate stimulus frequency. This difference disappears at a low stimulus frequency. Habituation was more pronounced as either the stimulus frequency increased or stimulus intensity decreased. Thus, our study demonstrates that habituation is socially regulated, and our model suggests that the socially mediate rate of habituation also depends on the characteristics (frequency and amplitude) of the stimulus inputs.

## Author contributions

CP, KC, FI, and SA performed research; CP, FI, and SA analyzed data; CP, FI, and SA designed research and wrote the paper.

### Conflict of interest statement

The authors declare that the research was conducted in the absence of any commercial or financial relationships that could be construed as a potential conflict of interest.
